# Treatment of Recurrent Optic Nerve Sheath Meningioma With a Secondary Course of Radiotherapy

**DOI:** 10.7759/cureus.17935

**Published:** 2021-09-13

**Authors:** Jibran A Sharieff, Andrew Melson, Ozer Algan

**Affiliations:** 1 Ophthalmology, University of Oklahoma Health Sciences Center, Oklahoma City, USA; 2 Radiation Oncology, University of Oklahoma Health Sciences Center, Oklahoma City, USA

**Keywords:** optic nerve sheet meningioma, onsm, radiotherapy, recurrent onsm, secondary radiotherapy

## Abstract

Optic nerve sheath meningiomas (ONSM) are benign neoplasms found surrounding the optic nerve that can affect vision, and potentially lead to blindness. The use of radiotherapy has been advocated to improve visual outcomes and minimize the risk of complications. We present a case of a 58-year-old woman who was treated with a second course of radiotherapy 27-years after initial radiotherapy for recurrent ONSM. The patient responded well to the second course of radiotherapy with good clinical and visual outcomes. This case report supports evidence that treatment with radiotherapy can improve visual outcomes in patients with recurrent ONSM with mild to moderate re-irradiation-related side effects.

## Introduction

Optic nerve sheath meningiomas (ONSM) are benign neoplasms of the arachnoid cap cells of the meninges around the optic nerve and account for about 2% of orbital tumors [1,2]. They are slow progressing tumors that can present with a classic triad of vision loss, optic atrophy, and optociliary shunt vessels, although this presentation is rare [3,4]. The degree of presentation is highly variable but the most common symptoms are vision loss, visual field defects, proptosis, and strabismus [1]. Without treatment, these tumors can often progress to blindness [2,3].

## Case presentation

A 58-year-old woman with a history of Chiari-type 1 malformation initially presented in 1993 with complaints of headache, dizziness, and blurry vision in her right eye (OD). On initial neuro-ophthalmological examination, she was found to have a best-corrected visual acuity of 20/15 in both eyes (OU). Despite preserved central acuity, she was found to have a dense relative afferent pupillary defect (RAPD), decreased color vision, and significant inferior arcuate scotoma in the OD. No abnormalities were noted in her left eye (OS). MRI and high-resolution CT imaging demonstrated enhancement of the right optic nerve within the orbit consistent with an ONSM. Given the preserved central visual acuity, the patient elected for close observation. On subsequent visits, the patient was found to have a progressive decline in visual acuity (20/50 OD) with significant visual field loss resulting in vision in the superior nasal quadrant only. Secondary to progressive vision loss, the patient elected to proceed with radiotherapy (RT1). She was treated with a single lateral conformal field to the right retro-orbital region to a dose of 4005 cGy given in 15 fractions (267 cGy per fraction) over three weeks. The treatment fields included a portion of the posterior retina to encompass the optic nerve enlargement extending up to the globe. A 5◦ gantry angle was used to avoid irradiating the contralateral optic nerve. The patient tolerated the treatment well. Following completion of RT1, the patient reported progressive improvement in vision and was found to have 20/15 vision OD with a full visual field and resolved RAPD. Surveillance MRI following radiotherapy showed a decline in the size of ONSM.

**Figure 1:**
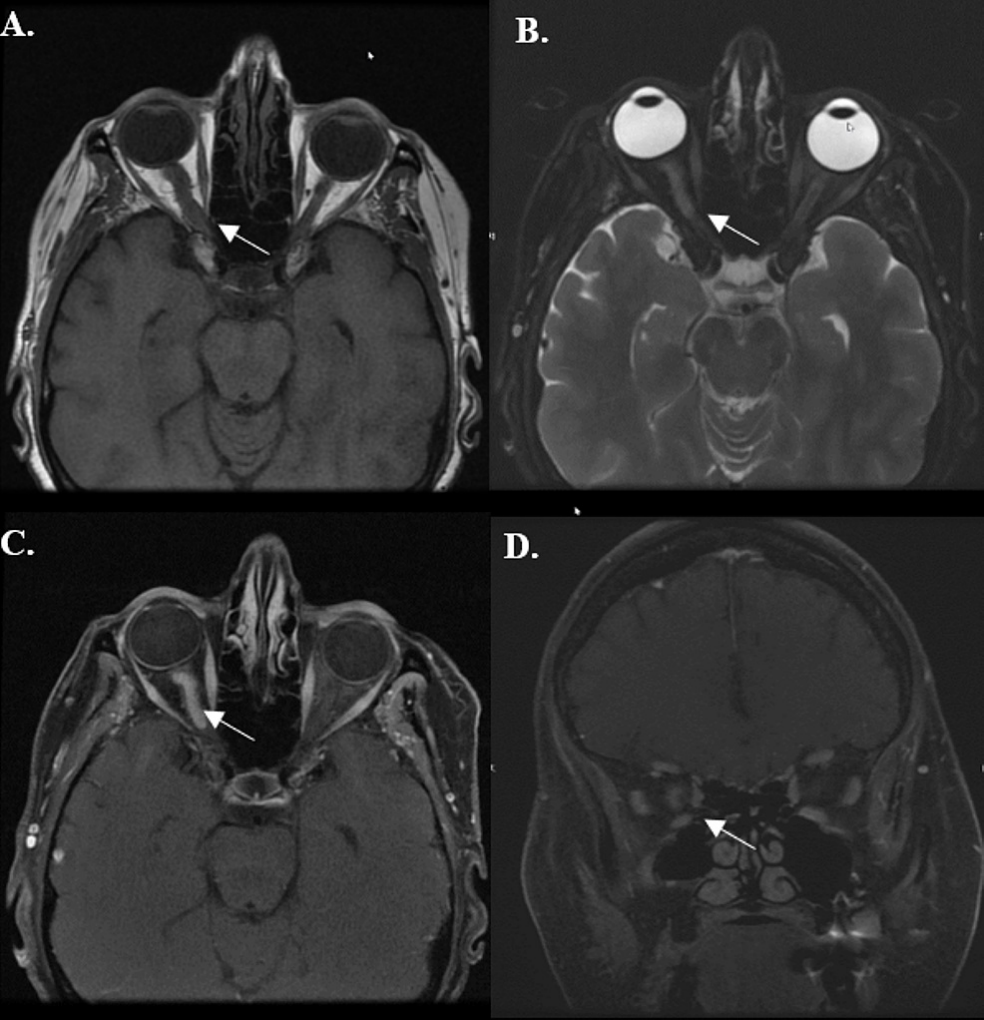
MRI images of the orbits one month prior to the start of second course of radiation treatment (RT2). A) Axial T1 fast spin echo (FSE) sequence. B) Axial T2 FSE sequence, demonstrating diffuse T2 hyperintensity along the intraorbital segment of the right optic nerve. C) Post-contrast axial T1 FSE sequence, demonstrating enhancement along the intraorbital portion of the right optic nerve. D) Post-contrast coronal FSE sequence, demonstrating near circumferential involvement of the right optic nerve by optic nerve sheath meningiomas (ONSM).

Nineteen years after the first treatment, the patient reported increasing visual shadows and a subjective decrease in vision in the OD. An MRI revealed a new enhancement around the right optic nerve consistent with type 1 ONSM recurrence contained within the orbit. With preserved central acuity, the patient elected again for close observation. The patient was closely followed for seven years following the initial recurrence of the tumor over which time, the peripheral field slowly but progressively declined to near-complete restriction and her RAPD returned. Visual acuity declined to 20/50 (OD). MRI at that time revealed diffuse T2 hyperintensity and contrast enhancement throughout the intraorbital segment of the right optic nerve-sheath complex (Figure 1). This was felt to represent an infield recurrence, although the initial treatment fields were not available for review. The patient's case was discussed in a multidisciplinary tumor board, and it was decided to proceed with re-irradiation prior to considering more invasive procedures.

**Figure 2:**
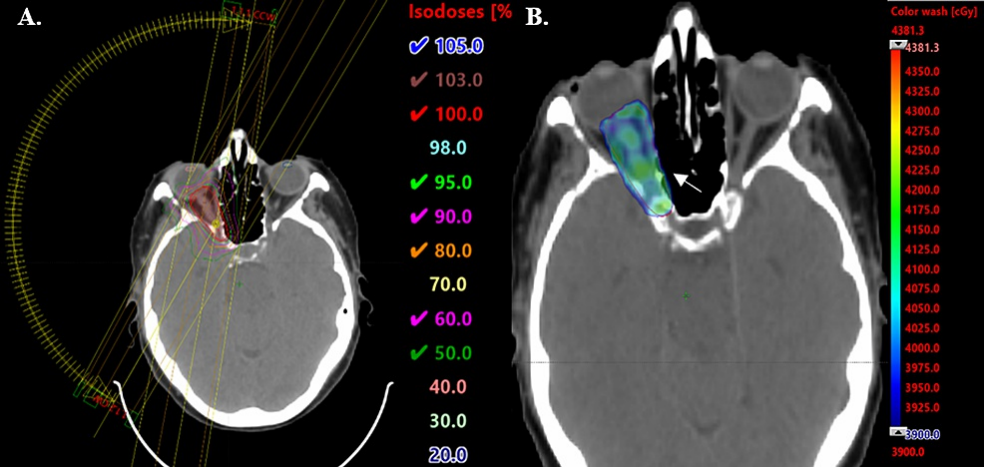
A) Axial view of treatment planning CT scan demonstrating partial arcs and the irradiated region. Isodose lines ranging from 50% to 105%. Field arrangement allows for significant reduction of radiation dose to the right orbit, pituitary gland, optic chiasm (not visualized), and contralateral optic nerve and orbit. B) Color wash showing doses above 3900 cGy. Light blue represents the prescription dose of 4000 cGy given in 16 fractions.

Because of the excellent response to the initial course of therapy, it was decided to proceed with a similar dose fractionation regimen for the second course of radiation treatment (RT2). Treatment planning CT scan was performed using a helical scanner with 0.125mm slice thickness and fused to the diagnostic MRI sequences. The gross tumor volume (GTV) was defined as the area of abnormality seen on the diagnostic MRI and encompassed all of the intra-orbital and most of the intra-canalicular portion of the optic nerve. An additional clinical target volume (CTV) margin for sub-clinical disease spread was not added. A planning target volume (PTV) expansion of 3-5mm was used to account for uncertainties in patient setup. Optimization structures for the PTV and the optic chiasm were created to limit the radiation dose to the pre-chiasmatic nerve fibers, so as to not affect the vision in the contralateral eye. Other organs at risk (OARs) included the bilateral orbits and the contralateral optic nerve. A treatment plan utilizing two partial arcs (from 210 degrees to 10 degrees in the clockwise and counterclockwise direction) and intensity-modulated radiotherapy treatments (IMRT) was used (Figure 2). The patient was re-irradiated to a dose of 4000 cGy given in 16 fractions. Doses received by the PTV and OARs are shown in Table 1. A thermoplastic mask was utilized to assist with patient positioning and the patient was instructed to look straight ahead prior to the treatment planning scan as well as prior to each treatment. Similarly, orthogonal pair kV imaging was used prior to each treatment to confirm the patient setup.

**Table 1:** Radiation dosages of planning target area and surrounding organs at risk. OAR - Organ at risk; Dmax – Maximum dose received by the structure; Dmean – Average dose received by the structure; D95 – Dose received by 95% of the structure; D5 – Dose received by 5% of the structure. Optic chiasm opti – optimization structure is similar to the optic chiasm but excludes a portion of the pre-chiasmatic optic nerve fibers on the involved right side.

Target/OAR	Dmax (cGy)	Dmean (cGy)	D95 (cGy)	D5 (cGy)
PTV	4381	4074	4000	4155
Right Orbit	4185	1575	198	3979
Right Optic Nerve	4312	4072	4010	4147
Right Lacrimal Gland	692	164	77	334
Right Lens	845	657	559	761
Optic Chiasm Opti	988	484	284	720
Pituitary Gland	1902	1182	900	1522
Left Orbit	1463	661	173	1108
Left Optic Nerve	837	545	380	729

**Figure 3:**
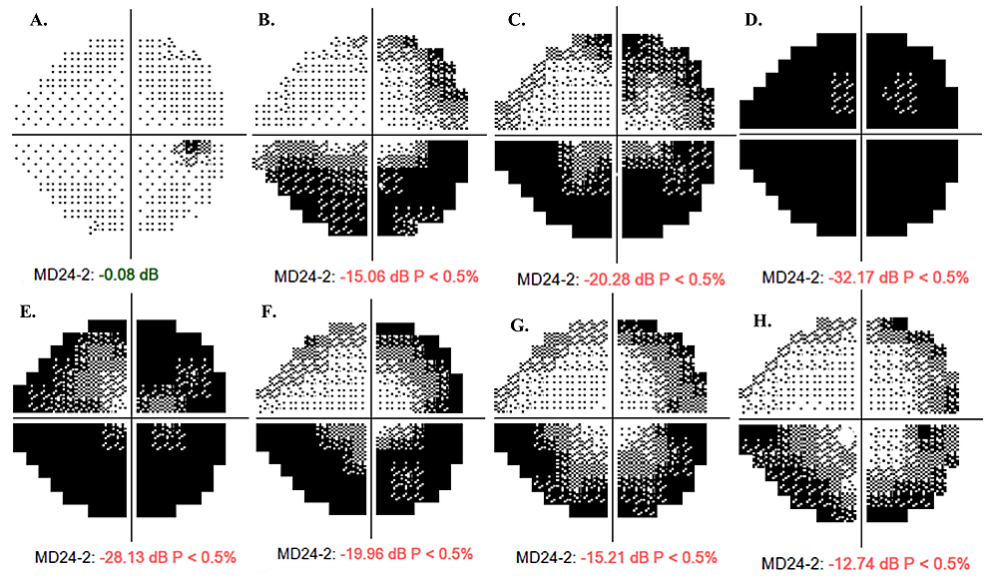
Right eye (OD) visual field progression overview. Visual field prior to treatment is represented by A-D; July 2013, July 2016, February 2017, and March 2020. Visual field post-radiation therapy is represented by E-H; May 2020, August 2020, December 2020, and March 2021.

Following RT2, the patient again noted progressive improvement in visual function and reported that she was no longer colliding with walls. Her visual acuity remarkably improved to 20/15 OU with an expansion in the superonasal and temporal visual fields to 71% of normal function (Figure 3). Following RT2, the patient experienced dry eye, transient dyschromatopsia, ocular neuromyotonia, and discomfort behind the eye. At the most recent follow-up, one year after the second radiotherapy course, the patient demonstrated lasting treatment effect with 71% of her normal visual field function and no evidence of radiation optic neuropathy/retinopathy. Retro-orbital discomfort was successfully treated with carbamazepine, which has similarly reduced the frequency of diplopia from ocular neuromyotonia. Her dry eye was well managed with ophthalmic drops. 

## Discussion

Diagnosis of an ONSM is difficult due to the variable presentation and slow-growing nature of the tumor. The best method for diagnosis is through imaging of the orbit and the cranium. MRI is superior to other modalities such as CT or ultrasound due to higher quality soft-tissue contrast allowing for differentiation between OSNM and other orbital tumors [1,5]. CT imaging can be used for purposes of identifying calcifications and bony anatomy to allow for a better understanding of the tumor dimensions in relation to patient anatomy. Imaging can identify three types of growth patterns of an ONSM: diffuse, globular, and fusiform [5]. Additional findings are smooth tumor margins and “tram-tracking” [1,6]. This is due to the meningioma's hyper-dense regions on either side of the optic nerve and is a classical sign for an ONSM [1]. Due to the location of the tumor, an incisional biopsy is rarely indicated as it carries a high risk of vision loss [1,6]. The role of biopsy is typically reserved for cases where conservative measures are not possible, such as in cases of aggressive disease course or in abnormal radiologic findings [6]. However, we acknowledge that without a biopsy of the tumor, the diagnosis of an ONSM for this patient cannot be proven with absolute certainty. Another diagnosis, such as lymphoma, would also be a possibility, especially in light of the rapid response that the patient had to her re-irradiation. However, the overall presentation and the time course of her disease process spanning over 27 years does not quite fit with the diagnosis of lymphoma.

Current methods of managing ONSM include observation, radiotherapy, or surgical resection [6]. Due to the high risk of blindness, if untreated, observation is not recommended [2,3]. ONSM is best managed with radiotherapy as outcomes are more often favorable with stability achieved in more than 80% of patients [6-9]. There are multiple forms of radiotherapy - fractionated external beam, three-dimensional conformal (3D-CRT), and IMRT [10]. It has been observed that there is no statistical significance in the outcomes and complications in patients based on the type of radiotherapy used to treat OSNM [1]. Total radiation doses are limited to less than 5400 cGy over 30 fractions to avoid causing radiation injury to the optic nerve [11]. Toxicity to the optic nerve has been documented and shows that this dose range is safe using modern radiation techniques [8]. A study done by Farzin et al. showed that in 140 patients in which neuro-optic structures such as the optic nerve or optic chiasm were in the field of treatment for meningiomas, the predominant symptoms post-radiation were limited to dry eye and cataract development [12]. Saeed et al. reported complications of radiation therapy in terms of early and late presentation [9]. Early-stage complications patients may encounter are temporary erythema, alopecia, and headaches whereas late-stage complications were dry eye and cataract development [9]. Rarely, patients may develop radiation-induced optic neuritis (RION) or mild radiation retinopathy [9,12]. Many studies support radiotherapy as the best method of management for ONSM and report that patients typically tolerate dosages less than 5400 cGy [8,11,13].

During our literature review, we did not encounter any literature utilizing re-irradiation for ONSM. Repeat radiation therapy has been used to treat other tumors of the brain. A retrospective study of patients with craniopharyngiomas showed that three out of four patients treated with repeat radiation treatment were able to improve or stabilize vision [14]. The study showed that most patients (75%) did not experience any significant changes to their endocrine function, despite previously developing endocrine complications from their first radiation therapy. Increased time between the first and second radiation therapy was recommended to allow for the tissues to repair from subacute radiation damage. Foran et al. concluded that re-irradiation therapy can be safe and effective for tumor control, however, they also recognized that long-term follow-up is necessary [14].

## Conclusions

In conclusion, we report a unique case report of a patient developing progressively worsening visual symptoms and undergoing re-irradiation 27 years after the initial course of radiation therapy for ONSM. This report suggests that irradiation was able to reduce the patients worsening visual symptoms and allowed for the preservation of vision with mild to moderate re-irradiation-related side effects. Further studies are needed to better establish the duration of effect, response rates, and the general safety profile associated with the second course of radiation for ONSM.
